# Empirical analysis of drought-induced cattle destocking in South Africa

**DOI:** 10.4102/jamba.v16i1.1557

**Published:** 2024-03-30

**Authors:** Prince Nketiah, Herbert Ntuli

**Affiliations:** 1 Department of Agricultural Economics, Extension and Rural Development, Faculty of Agriculture, University of Pretoria, Pretoria, South Africa

**Keywords:** drought, cattle destocking, smallholder cattle farmers, standardised precipitation index (SPI), South Africa

## Abstract

Destocking as a drought mitigation strategy exposes smallholder cattle farmers to adverse effects, including the distortion of farm planning and income loss, as cattle are sold off regardless of the market price. Factors influencing destocking as a drought mitigation strategy for smallholder cattle farmers have received less attention in the literature. The study assessed the relationship between drought and cattle destocking as well as factors that affect farmers’ destocking decision. The relationship between drought and cattle destocking was assessed using correlation analysis, while determinants of destocking were identified through the zero-inflated Poisson (ZIP) regression model, which controlled for structural zeros. The research covered the period 2008–2017 using secondary data from the National Income Dynamics Study (NIDS), the South Africa Weather Service and the Food and Agriculture Organisation (FAO). The study found that drought has direct correlation with the quantity of beef produced in South Africa at −0.67, with a 1% significance level. Farmers’ socioeconomic characteristics such as cattle herd size, income, secondary occupation, fodder purchase and ownership of land positively influenced cattle destocking decision while household size and cattle loss during drought influenced destocking decision negatively.

**Contribution:**

The study estimated the determinants of smallholder cattle farmers’ decision to destock during drought, using a count model and accounted for socioeconomic and farmer-specific factors.

## Introduction

Effective resource allocation and planning of smallholder agriculture depend on weather variables such as humidity, drought, temperature and rainfall in addition to institutional and socioeconomic variables including social network, income and experience (Mabe, Nketiah & Darko 2014). To the farmer, being forearmed with relevant weather updates directly influences the day-to-day decisions and helps to cater for expected events (Craft 2001). Drought is a major weather phenomenon that has adverse effects on agriculture planning and management. Moreover, drought poses a threat to livelihood asset holdings, hence making the strategies for disaster vulnerability reduction essential for farmers, a stance upheld by the Sendai Framework for Disaster Risk Reduction (UN 2015). A public initiative to identify vulnerable locations and groups affected by disasters has been proposed to governments and nongovernmental agencies to assist in finding lasting mitigation and adaptation measures (OECD 2009).

An essential core of the drought risk to beef cattle production and the livelihood of cattle farmers is the establishment of an actual decline in cattle herd size. This subject has attracted some level of research through which the results evidence a significant reduction in cattle herd size during drought occurrence (Dzavo et al. 2019; Kanwal, Smita & Prem 2020; Maluleke, Tshabalala & Barkhuizen 2020; Ngaka 2012; Oba 2001; Vetter, Goodall & Alcock 2020). The decline could be because of other factors, including the loss of cattle to drought stresses or the managerial strategy of destocking by cattle farmers to navigate the menace of drought. This study focuses on the latter to shed light on the plight of smallholder farmers’ short-term drought management decisions, which include selling off cattle regardless of the going market price. The study provides vital information for livelihood enhancement in the livestock sector. It contributes towards the achievement of Sustainable Development Goal (SDG) 1 and SDG 2, which seek to bridge economic inequality among citizens and eradicate hunger, respectively. These SDGs are also in line with the South African National Development Plan (SANDP). This study seeks, firstly, to examine the relationship between drought and cattle destocking and, secondly, to estimate factors that influence destocking decision as a mitigation strategy for smallholder cattle farmers during sustained drought occasions.

The vegetational attribute of South Africa reflects the great potential for breeding livestock and ruminants such as cattle, goats and sheep. The potential is a viable livelihood source for commercial and smallholder investment. Cattle herd size among emerging and communal farmers averages 19 per household against 413 for commercial farms (Scholtz et al. 2008). Their study further revealed that smallholder cattle farming aims to raise cattle for the market rather than keep them for farm draught power and dairy purposes. Moreover, a minority of farmers in South Africa are engaged in smallholder agriculture and food production, unlike other African countries within the sub-Saharan region. Communal and emerging smallholder farmers contribute 40% of the cattle farming population in the country (Louw, Louw & Flandorp 2018). To promote equity in and access to resource ownership in South Africa, government policy since 1994 has aimed at redistributing land to the resource poor. Farmers who benefit from this policy are referred to as emerging farmers.

Smallholder cattle farming relies mainly on forage pasture, which is easily accessible, convenient and cheap. Cattle farmers found in developed and emerging economies mainly operate at a smallholder level and constitute a vital front in the provision of food, raw materials and landscaping (FAO 2019). The feeding system that allows cattle to forage outside confinement is termed an extensive or pasture fed (Oduniyi, Rubhara & Antwi 2020). Also, they emphasised that smallholder farmers mostly rely on this type of system to satisfy the nutritional requirements of their herd. The study of constraints and adaptive capacity of smallholder cattle farmers in South Africa (Oduniyi et al. 2020) highlighted an overlap between intensive and extensive systems of housing for cattle, and it is known as the semi-intensive system where cattle are housed in a confined area at certain times of the day and released to forage at another time. During drought, resource-constrained farmers face the difficulty of providing adequate fodder for their herds. Mitigation strategies against drought help in planning and management decisions that aim at reducing the impact of hazardous events (Keshavarz, Ezatollah & Kamgare-Haghighi 2010). On-farm strategies such as grazing adjustment, fodder purchase and feed rationing are some short-term mitigation measures that farmers engage in to navigate feeding stress. In contrast, others sell off some cattle in their quest to navigate the effect of drought on their livelihood (Salmoral, Ababio & Holman 2020).

The severity of the impact of a particular drought determines the response by livestock farmers (Haigh et al. 2019). Nonetheless, the importance of tracking the behavioural patterns of cattle farmers towards mitigation of drought cannot be underscored enough. Such insight and knowledge from drought risk assessment will help in achieving essential milestones in the planning and management of drought impact on the livelihood of smallholder cattle households. Cattle farmers confronted with the difficulty of drought-induced feed shortages may consider unincentivised destocking in order to offset short-term challenges (Bahta 2020, Toulmin 1985). Destocking allows for optimisation of feed rations aimed at enhancing the performance of remaining herds during periods of unfavourable conditions such as drought.

On the other hand, incentivised destocking is sometimes carried out by developmental agencies as a means of critical intervention for saving livestock farmers’ livelihood during severe drought periods. CARE Ethiopia undertook such externally motivated intervention in the Oromiya region of southern Ethiopia during the drought period in 1999 (Tieke 2003). CARE Ethiopia provided cattle farmers with grains in exchange for weak but healthy cows. The purchased animals were processed into dried meat and distributed to charity towards the improvement of malnutrition among the young and vulnerable in society (Morton & Barton 2002; Tieke 2003). Similarly, Vétérinaires Sans Frontières Belgium (VSF-Belgium) undertook a destocking intervention for the 2005 drought in north-western Kenya (Watson & Binsbergen 2006). Research on unincentivised destocking as a drought mitigation strategy for smallholder cattle farmers has received less attention in the literature. This study seeks to fill the gap by contributing to the frontiers of smallholder livelihood development and sustainability.

### Conceptualisation of study

Southern Africa has recorded natural disasters in recent times (Owusu-Sekyere, Lunga & Karuaihe 2021). The 2022 World Risk Index reports that Southern African states are vulnerable to natural disasters such as floods and drought (Hilft 2022). South Africa ranks among the top 15 African countries susceptible to extreme weather events (Hilft 2022). Naturally occurring incidences such as disease and extreme weather variations, among others, hinder farmers’ livelihood sustainability. The risk of drought is natural and catastrophic in that it falls into a category that the OECD (2009) describes as having the characteristic of a low occurrence frequency but high losses in terms of damage caused.

Agricultural uncertainties and risks come in different dimensions and scales, including those that arise from natural causes. Adverse weather conditions are hypothesised in the literature to pose livelihood risks to smallholder cattle producers in maximising the potential benefit of their herd and sustaining their livelihoods (Dzavo et al. 2019). Disaster vulnerability measurements are based on exposure, susceptibility, coping and adaptive capacities.

This study investigated the coping strategy of smallholder cattle households towards drought shock. Response strategies remain a critical component of farm management practices. Such strategies are expected to help mitigate the negative impact that may arise from drought events on farmers’ livelihoods and investments.

The drought effect on smallholder cattle development is such that it hampers the expected growth rate of cattle and sometimes results in the death of some cattle within the herd. This also has a direct effect on the expected incomes of the farmer. Some farm households may have the capacity to acquire feed alternatives such as grains to protect their investment. The demand and supply dynamics in drought will affect the sale of cattle or purchase of feed. A drought-induced price fall for cattle and a price hike for grains go against the farmer’s welfare. On the other hand, the sale of cattle is a plausible means by which farmers save to reinvest later. Losing cattle to extreme weather events would lead to undesirable outcomes such as asset base decline, reduced household incomes and welfare.

## Research methods and design

### Data

The data for this study were sourced from the National Income Dynamics Study (NIDS), the South Africa Weather Service (SAWS) and the Food and Agriculture Organisation (FAO). The NIDS survey and weather data covered all the administrative provinces of South Africa in five cross-sections from 2008 to 2017. Data on yearly beef output were sourced from FAOSTAT, while the SAWS provided the 12-month standardised precipitation index (SPI) data. As shown in Table 1, the 2017 survey recorded 21% out of 311 households that engaged in the destocking of cattle during the drought periods of 2017.

**TABLE 1:** Distribution of cattle destocking of households.

Year	Frequency	Total households	Percentage
2008	37	385	9.6
2012	32	205	15.6
2015	42	379	11.1
2017	64	311	20.6

Ecologically, South Africa comprises largely semi-arid and arid zones, with significant parts of the country experiencing summer rains and relatively dry winter periods. The country covers a total of about 122.3 million hectares of land surface, of which about 80% is suitable for grazing (ed. DAFF 2018). The Agricultural Sector Education and Training Authority (2020) (AgriSETA 2020) highlights South Africa’s contribution of about 24% of total beef on the African continent, representing 1% of global beef production. The beef industry takes a share of about 34.1% of total agricultural output domestically. The significant contribution of the beef sector of South Africa to the world economy makes the sector viable and one that is essential for smallholder livelihood enhancement.

### Standardised precipitation index

This study used the SPI in the measurement of drought as shown in Table 2. Precipitation records in South Africa from 2008 to 2018 from different weather stations located within the country were transformed into a normal distribution, which ranges from 3 to −3 (Hayes et al. 1999). The transformed values are referred to as the SPI.

**TABLE 2:** Standardised precipitation index classifications.

Standardised precipitation index	Drought category
2.00 and above	Extremely wet
1.50 to 1.99	Very wet
1.00 to 1.49	Moderately wet
0.50 to 0.99	Mild wet
0.49 to − 0.49	Normal
−0.50 to − 0.99	Mild dry
−1.00 to − 1.49	Moderately dry
−1.50 to − 1.99	Severely dry
−2.00 and below	Extremely dry

*Source*: Adapted from Adnan, S., Kalim, U., Shouting, G., Ashfaq, H.K. & Ziqian, W., 2017, ‘Shifting of agro-climatic zones, their drought vulnerability, and precipitation and temperature trends in Pakistan’, *International Journal of Climatology* 37(S1), 529–543. https://doi.org/10.1002/joc.5019

In the context of this study, SPI variations are expected to have a significant relationship with total beef output in South Africa across the period under study. A negative SPI would imply that drought occurred in the particular year, while a positive index would imply otherwise depending on total precipitations recorded during the year.^1^

Following Negassa et al. (2012) and Duba, Solomon and Tegegne (2021) on pastoral farmers’ choice of coping strategies, variables such as off-farm income, age and gender, among others, were found to be significant. This study expands the model to include beneficiaries of social support, remittance, fodder purchase and loss of cattle as essential factors in livestock management practice during periods of shock. The decision to destock during drought is hypothesised to be affected by the asset base of the farm household. Smallholder farmers’ asset was captured as land ownership, as is familiar to most farmers. Definitions of the variables considered in this study are shown in Table 3.

**TABLE 3:** Definition of variables.

Variable	Definition	Measurement	Apriori
Herd size	Total number of cattle per herd	Count	NA
Income	Total household monthly income	Amount (Rand)	±
Education	Years spent in school by farmer	Count	±
Age	Years of household head	Count	±
Crop household	Households having crop farm	1 = Yes	±
		0 = No	±
Remittance	Households receiving remittance	1 = Yes	±
		0 = No	±
Social support	Households receiving social support	1 = Yes	±
		0 = No	±
Cattle loss	Households experience cattle death	1 = Yes	±
		0 = No	±
Secondary job	Household head having secondary off-farm occupation	1 = Yes	±
		0 = No	±
Fodder	Household purchase fodder	1 = Yes	±
		0 = No	±
Household size	Total number of people living in the household	Count	±
Land ownership	Total cultivated and uncultivated land owned by household	0 = No land 1 ≤ 5000 m^2^ 2 ≤ 5000 m^2^	±

### Zero-inflated Poisson model

To gain insight into smallholder cattle farmers destocking during severe drought, the study employed a zero-inflated Poisson (ZIP) model. Smallholder cattle farmers’ decision to destock is the dependent variable *D_i_*. It takes on a count, indicating how many cattle an individual farmer chooses to destock. Poisson regression is appropriate for analysing count-dependent variables (Greene 2003). The general econometric specification of the Poisson model is expressed as:

$$P_{r}\left(D_{i}=d_{i}|x_{1}\right)=\frac{\pi^{d_{i}}e^{-\pi }}{d_{i}!},d_{i}=0,1,2,3\ldots$$ [Eqn 1]

The underlying assumption of the Poisson model is that it relies on equality between the mean and variance. A violation of this assumption occurs when there is overdispersion (i.e., variance greater than mean), as is the case in this study; hence an estimation of the generic Poisson model would yield inefficient estimates (Cameron & Trivedi 2010). Factors that may account for the overdispersion are mainly because of excess zeros that arise from either temporal dependency or abstention. For example, temporal dependency may arise when market prices hinder a farmer’s decision to sell. On the other hand, abstention may result from farmers’ unwillingnes to sell off cattle to preserve prestige or cultural values. Such structural zeros need to be accounted for to avoid the production of spurious estimates.

Zero-inflated models independently set a distribution for excess zeros, assuming that zeros are generated by a separate process rather than the count values. Based on the result from Akaike’s information criterion (AIC) and Bayesian information criterion (BIC), the ZIP was most suitable for this study compared to the generic Poisson model and negative binomial model. Equation 2 shows the first stage of the logit process that predicts excess zeros. The two-part ZIP model has the zeros censored in the second stage count estimation and can be defined as:

$$P_{r}=(y_{i}=0)=\lambda +(1-\lambda )e^{-x}$$ [Eqn 2]

$$P_{r}=(y_{i}=d_{i})=(1=\lambda )\frac{\pi^{d_{i}}e^{-\pi }}{d_{i}!},d_{i}>1$$ [Eqn 3]

where outcome *y_i_* takes on non-negative integer values; *d_i_* represents the expected count of cattle destocked by the *i^th^* farmer during drought; λ is the probability of excess zeros. The mean and variance are represented by (I – λ)^π^ and π (I – *λ*) (I + *λ*), respectively.

### Ethical considerations

Ethical clearance to conduct this study was obtained from the University of Pretoria, Faculty of Natural and Agricultural Sciences Ethics Committee (No. NAS061/2023).

## Results and discussion

The results of the continuous variables included in this study are presented in Table 4. The age of the household head averaged 62 years, indicating that these households are led by the late middle age class who might have considerable experience with drought occurrence in South Africa. Household size, on the other hand, averaged six members per household. The average monthly income was about 7248 South African Rands, while the average herd size and education were eight cattle heads and 6 years of schooling, respectively.

**TABLE 4:** Descriptive statistics of continuous variables.

Variable	Mean	SD
Age (years)	61.77	14.99
Household size	5.83	3.72
Income (Rand)	7 247.81	5 608.26
Herd size	8.06	7.19
Education (years)	6.16	4.25
SPI	−0.11	0.37
Beef output (mt)	944 048.8	101 017.9

SPI, standardised precipitation index; SD, standard deviation.

Out of the 311 smallholder cattle households captured in the survey, female-led cattle households (59%) were more than male-led households (41%) as shown in Table 5. A limited number of farmers engaged in fodder purchase and secondary off-farm jobs, representing 18% and 11%, respectively. Also, 21% of the farmers experienced widespread cattle death, whereas 35% were remittance receivers. Farmers that owned land area less than 5000 m^2^ comprised 68% of respondents as against 16% who owned land greater than 5000 m^2^. Twenty-one percent of the smallholder cattle households owned no land.

**TABLE 5:** Descriptive statistics of dummy variables.

Variable	Category	Frequency	Percentage
Gender	Female	185	59.49
	Male	126	40.51
Destock	No	246	79.35
	Yes	64	20.65
Remittance	No	201	64.63
	Yes	110	35.37
Secondary income	No	275	88.42
	Yes	36	11.58
Social support	No	68	21.86
	Yes	243	78.14
Fodder purchase	No	254	81.94
	Yes	56	18.06
Cattle loss	No	244	78.71
	Yes	66	21.29
Land ownership	No land	64	20.58
	< 5000 m^2^	198	63.67
	> 5000 m^2^	49	15.76

### Relationship between drought and destocking

**FIGURE 1:**
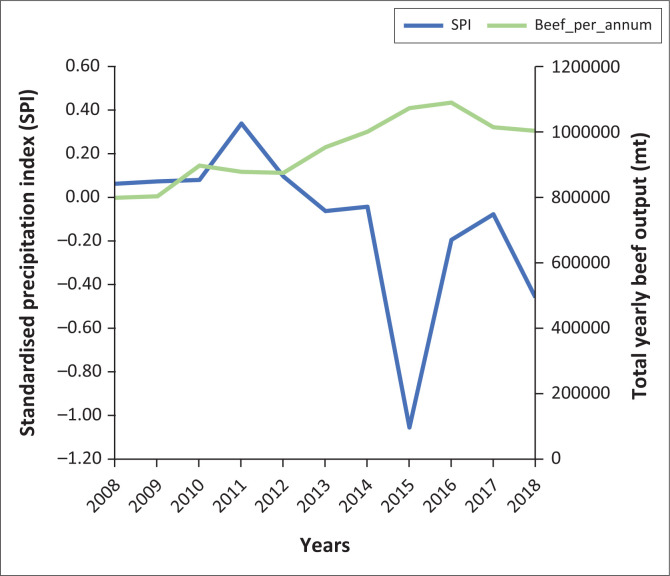
Standardised precipitation index and beef output for South Africa from 2008 to 2018.

The relationship between SPI and total beef outputs of South Africa from 2008 to 2018 is presented in Figure 1.

### Standardised precipitation index and beef output for South Africa from 2008 to 2018

The figure shows a negative relationship between drought and total beef outputs such that drier years correspond with increased beef output and vice versa. Correlation analysis (Table 6) between SPI and beef output between 2008 and 2018 shows a −0.67 coefficient; this implies that drought (low SPI) directly corresponds with the quantity of beef produced in South Africa. It can be observed that there has been a steady rise in beef output from 2013, simultaneously with a below 0.00 SPI indices till 2016, where South Africa’s highest beef output within the decade under review was recorded. The peak observation of beef output succeeds the severe drought of 2015 as an ex-post effect. The drought event 2015 is considered the severest in the country’s history since 1904 (Mare, Yonas & Van Niekerk 2018).

**TABLE 6:** Correlation matrix of standardised precipitation index and beef output.

Variables	SPI	Beef output
SPI	1.00	-
Beef output	−0.669*** †	1.00

SPI, standardised precipitation index.

† , *p*-value = (0.024).

*** , 1% level of significance.

On the other hand, analysis of the 2017 NIDS cross-sectional data corroborates with beef output, as the percentage of households engaged in cattle destocking was highest in 2017 (Table 1). This finding is in line with the study of Toulmin (1985), which highlighted pastoralist adoption of destocking as a drought mitigation strategy. Smallholder cattle farmers contribute about 25% to the total beef output in South Africa (Mmbengwa et al. 2016). Risk-averse smallholder cattle farmers may engage in this strategy to protect their investments in cattle farming.

### Determinants of farmers’ destocking decision

The output from the ZIP regression (Table 7a and Table 7b) indicated that variables such as cattle herd size, monthly income, record of cattle loss, land ownership, household size, fodder purchasing and household with secondary occupation significantly determine the cattle destocking decision of smallholder cattle farmers during drought. The results from the Poisson and negative binomial models showed similar significant estimates for household and herd sizes, gender and income. Even though the two models recorded fewer significant variables compared to the ZIP model, the negative binomial model had the least. The interpretation of the explanatory variables was based on the incidence rate ratio (IRR) and odds ratio (OR), which are derived from taking exponents of the parameter estimates of the ZIP. Incidence rate ratio measures the magnitude by which an independent variable affects the movement from the destocking of a single cow to that of multiple cattle among smallholder cattle farmers. Incidence rate ratio and OR greater than one will imply that the event has a higher probability of happening than the control group and vice versa.

**TABLE 7a:** Estimation results of farmers’ destocking decision.

Variable	Poisson			Negative binomial			Zero-inflated Poisson		
	Coef.	S.E.	IRR	Coef.	S.E.	IRR	Coef.	S.E.	IRR/OR
Gender	0.477***	0.187	1.611	0.508**	0.255	1.661	0.289	0.238	1.335
Education	0.044	0.028	1.045	0.063*	0.036	1.065	0.040	0.032	1.041
Log_age	0.078	0.453	1.081	0.232	0.563	1.261	0.134	0.521	1.143
Log_income	0.608***	0.158	1.837	0.708***	0.208	2.029	0.796	0.219	2.216***
Log_hhsize	−0.513***	0.152	0.599	−0.574***	0.213	0.563	−0.707	0.174	0.493***
Remittance	−0.315	0.214	0.730	−0.388	0.297	0.679	−0.324	0.253	0.723
Secondry_job	−0.005	0.281	0.995	−0.224	0.413	0.799	0.602	0.319	1.827*
Log_herd_size	0.969***	0.129	2.636	1.008***	0.179	2.739	0.497	0.216	1.644**
Cattle_loss	0.226	0.229	1.253	0.176	0.323	1.193	−0.617	0.311	0.540**
Fodder_purchase	−0.611**	0.274	0.543	−0.550	0.507	0.577	0.987	0.406	2.684**
Land < 5000 m^2^	−0.467**	0.224	0.627	−0.371	0.325	0.690	−0.287	0.304	0.750
Land > 5000 m^2^	0.300	0.272	1.349	0.549	0.429	1.732	0.856	0.351	2.355**
_cons	−8.039***	1.867	0.000	−9.735***	2.694	0.000	−8.062	2.291	0.000***
Alpha	-	-	-	1.486	0.466	-	-	-	-

IRR, incidence rate ratio; OR, odds ratio; S.E., standard error.

* , *p* < 0.1;

** , *p* < 0.05;

*** , *p* < 0.01.

**TABLE 7b:** Estimation results of farmers’ destocking decision.

Variable	Poisson	Negative binomial	Zero-inflated Poisson
LR chi^2^ (12)	191.12	147.28	146.14
Prob(χ^2^)	0.000	0.000	0.000
Pseudo R^2^	−228.70	−209.99	−197.01
AIC	483.401	447.988	434.011
BIC	531.892	500.210	508.613

AIC, Akaike’s information criterion; BIC, Bayesian information criterion.

There is a positive and significant (1%) relationship between monthly income and the decision to destock. This shows that the likelihood of smallholder farmers destocking more cattle increases as income increases. It also shows that high-income earners are most likely to avert their perceived risk by selling off some cattle to invest in other off-farm ventures or save their capital to restock at a convenient period. Transferring risk as a way of mitigating against shocks has been highlighted in the literature (Hurst et al. 2012; Tadesse & Brans 2012).

On the other hand, smallholder cattle households’ engaging in secondary occupation is shown to have a direct relationship with the likelihood of engaging more cattle destocking during severe drought at 10% significance level. This result suggests that households that receive secondary off-farm income are not likely to cushion their farm investment with the financial benefit they get from secondary off-farm activities. Rather, they may engage in selling off cattle to mitigate against drought. A plausible reason for this observation may be that such cattle owners may not have adequate attention, and care for the herd because of competing schedules of work and farming, for that matter, may opt to destock during drought.

Cattle herd size had a positive and significant (5%) relationship with smallholder cattle farmers’ decision to destock during severe drought. This implies that an increase in herd size enhances the likelihood of smallholder households’ destocking more cattle during drought. This result shows that households with larger herd sizes are likely to destock more cattle to cope with drought pressures as fodder availability and access become a significant challenge for farmers. This is because the size of livestock is a key determinant of the choice of mitigation strategy for drought (Duba et al. 2021).

At a 1% level of significance, the negative relationship between household size and the decision to destock shows that there is less likelihood of destocking as household size increases. This may be attributed to the fact that larger household membership allows for consolidating resources such as labour, capital and intellect, among others, which can be marshalled to source alternative feed sources. This could go a long way to help mitigate the impact of drought on cattle. The positive relationship between smallholder cattle farmers’ fodder purchase decision and the decision to destock is significant at 1% and shows that farmers who purchase fodder within drought year are more likely to destock cattle during severe drought. This result reinforces and resonates that destocking allows for optimising feed rations for remaining herd during drought (Bahta 2020).

The result indicates that farmers who lost some of their cattle during the drought were less likely to opt for cattle destocking as a drought mitigation measure compared to those who did not lose cattle. This relationship is negative and significant at 5%, indicating that losing cattle discourages smallholder farmers from destocking and might be because smallholder cattle herd sizes may not be large enough (mean herd size equals eight in Table 4) to warrant additional destocking once natural causes have already taken a toll.

The result further indicates that the land size contributes to smallholder farmers’ destocking decision at a 5% significant level. The result indicates that smallholder cattle farmers’ ownership of land sizes greater than 5000 m^2^ increases their likelihood of destocking more cattle relative to farmer households that do not own land. This finding may imply that farmers with larger land sizes are most likely to have larger herd sizes and hence may destock more cattle to effectively mitigate against drought compared to farmers without large land sizes.

## Conclusion

The study found that drought (low SPI) corresponds inversely with the quantity of beef produced in South Africa, with cattle destocking being a plausible driver of such a relationship. This finding suggests that smallholder cattle farmers engage in destocking mainly as a risk transfer strategy. Adopting this strategy comes at a cost to the farmer, such that expected margins on cattle sales would either be differed or forfeited because of an increase in the supply of cattle to the market. The study further highlights that farmer socioeconomic characteristics such as cattle herd size, income, secondary occupation, fodder purchase and ownership of land influence cattle destocking decision during drought. This shows that resource-endowed smallholder farmers are more likely to engage in this risk transfer strategy than less-endowed farmers.

Therefore, smallholder farmers in South Africa should be encouraged to adopt suitable market tools in the form of micro-insurance to help protect their livelihood by providing a safety net for transferring risks. Financial institutions and other agricultural stakeholders should carve out this initiative to help sustain communal and emerging cattle farmers against the peril of periodic drought events. Lastly, future research should consider analysing smallholder cattle households’ welfare loss owing to a fall in market price during drought-induced cattle destocking.

## Footnotes

1 It is vital to note that a decrease in drought means an increase in SPI and the other way round. This implies that SPI parameter estimates will take opposite interpretations (e.g. a positive estimate shall be interpreted as a negative relationship vice versa).
